# The diabetic retina–brain axis hypothesis in diabetic cognitive impairment: from pathophysiological mechanisms to therapeutic implications

**DOI:** 10.3389/fendo.2026.1754543

**Published:** 2026-01-28

**Authors:** Luofan Zhang, Lerong Zhang, Haoyang Wen, Sixian Wu, Hanbo Yu, Lingyan Zhao, Guiping Li

**Affiliations:** 1 Department of Acupuncture and Moxibustion, First Teaching Hospital of Tianjin University of Traditional Chinese Medicine, Tianjin, China; 2 National Clinical Research Center for Chinese Medicine, Tianjin, China; 3 Department of Nephrology, First Teaching Hospital of Tianjin University of Traditional Chinese Medicine, Tianjin, China; 4 Research Center of Experimental Acupuncture Science, Tianjin University of Traditional Chinese Medicine, Tianjin, China; 5 School of Acupuncture-Moxibustion and Tuina, Tianjin University of Traditional Chinese Medicine, Tianjin, China

**Keywords:** diabetic cognitive impairment, neuroprotection, oxidative stress, retina-brain axis, therapeutic strategy

## Abstract

Diabetic cognitive impairment (DCI) is a frequent complication of diabetes that significantly reduces the quality of life of elderly patients and substantially increases the societal health–care burden. Recent epidemiological investigations have demonstrated that the severity of diabetic retinopathy (DR) is independently associated with a greater risk of cognitive dysfunction. To clarify the relationship between ocular and cerebral comorbidities in diabetes, this paper proposes the diabetic retina–brain axis hypothesis. By reviewing barrier dysfunction, neuroinflammation, and oxidative stress, we systematically present evidence that supports the involvement of the retina–brain axis in DCI and highlight the shared mechanisms that underlie retinal and cerebral damage in diabetes. Therapeutic strategies that target the retina–brain axis, such as hypoglycemic agents, antioxidants and neuroprotective interventions, provide benefits for retinal health and cognitive function. Investigating the mechanisms underlying this axis offers important insights for early diagnosis, prevention and treatment of DCI. Consequently, this research can guide the development of more effective interventions, for example by informing the use of sodium–glucose cotransporter 2 (SGLT2) inhibitors to protect both retinal microvasculature and neuronal integrity. Future research should prioritize elucidating the pathways of information transmission between the retina and the brain, and clarifying the molecular and cellular basis of these processes. This will provide theoretical support for the development of cross–organ collaborative protection strategies.

## 1 Introduction

Diabetes mellitus (DM) is a global metabolic disease, with an estimated 589 million people currently affected worldwide (1). While the harmful effects of chronic hyperglycemia are well-established, recent studies indicate that the natural course of diabetes is dynamically evolving. This trend is most evident in type 2 diabetes. Furthermore, in type 1 diabetes, advances in technology assisted care and disease-modifying strategies similarly redirect its disease trajectory. Consequently, through preventive measures, intensive lifestyle interventions, and emerging therapies, the overall clinical trajectory of diabetes is being actively reshaped. At the same time, diabetes simultaneously accelerates systemic aging and elevates the risk of age-related neurodegenerative diseases. Beyond the typical microvascular and neurological complications, diabetes is an important independent risk factor for cognitive impairment (2). Diabetic cognitive impairment (DCI)is mainly characterized by declines in memory, executive function, and attention, and is often accompanied by structural brain changes and abnormal neural activity. Numerous epidemiological studies have reported that diabetic individuals have a 2–3–fold higher risk of cognitive decline compared with non–diabetic subjects (3, 4). Against this background, as a consequence of diabetes with limited response to current treatments and a lack of systematic intervention strategies, early detection and intervention for DCI are increasingly crucial. Therefore, clarifying early diagnostic methods and establishing systematic intervention strategies may not only help delay its progression but also improve long-term neurocognitive outcomes for patients by positively influencing the overall course of diabetes.

The pathogenesis of DCI is complex and is generally attributed to cerebral alterations, blood–brain barrier (BBB) disruption, neuroinflammation, and lymphatic system dysfunction. However, these mechanisms do not fully explain the frequent coexistence of DCI with other diabetic complications. Diabetic retinopathy (DR) is a prevalent microvascular complication of diabetes, characterized by leakage and obstruction of the retinal microvasculature. Epidemiological evidence indicates that DR is independently linked to reduced cognitive function and an increased risk of dementia (5–7). For example, a longitudinal cohort study of 29 961 patients aged over 60 with type 2 diabetes found that those with retinopathy had a 42 % higher risk of developing dementia (8). This demonstrates a significant comorbidity between the two conditions. Research has shown that the topological organization of the brain in patients with DR is disrupted, suggesting that retinal microvascular abnormalities correlate with brain network dysfunction (9). Multimodal magnetic resonance imaging (MRI) further reveals that decoupling of functional and structural aspects of the visual network is closely associated with cognitive decline (10).

Anatomically and developmentally, the retina is a specialized extension of the central nervous system (CNS). Emerging from the forebrain during embryogenesis, it maintains a direct physical connection to the brain via the optic nerve (11). This shared origin is reflected in their striking structural and functional homology. Both organs share analogous features, including specialized barrier systems known as the BBB and blood-retinal barrier (BRB), dense neuronal networks, and supportive glial cells such as astrocytes and Müller cells (12). It is precisely these profound similarities that establish the retina as a unique and effective window for observing CNS pathology in a living patient. Retinal disorders not only reflect cerebral abnormalities but also have higher diagnostic value because they often appear earlier than corresponding brain changes. Under hyperglycemic conditions, both retina and brain may suffer concurrent damage through shared pathogenic mechanisms, notably disruption of the integrity of the BRB and BBB. This disruption leads to vascular leakage, tissue edema, capillary perfusion deficits, and neurovascular unit dysfunction. Such pathological processes are observable in the retina as well as in hippocampal subregions, prefrontal cortex, and other brain areas involved in cognition in diabetic patients.

In addition to microvascular lesions, DR and DCI share several common molecular pathogenic mechanisms. Hyperglycemia can trigger systemic inflammatory responses and oxidative stress, promote accumulation of advanced glycation end products (AGEs), and activate inflammatory pathways such as nuclear factor–κB (NF–κB), ultimately causing neuronal injury, glial cell activation, and synaptic dysfunction (12). Moreover, the progression of both DR and DCI is associated with metabolic disturbances, including insulin–signaling pathway abnormalities, mitochondrial dysfunction, and impaired autophagic flux (13). These findings indicate a potential pathological link between the two conditions at the molecular level. This supports the existence of a common pathogenesis that affects both retina and brain in diabetes, thereby reinforcing the diabetic retina–brain axis hypothesis.

Within this framework, the retina is not merely a passive target organ vulnerable to damage; its structural and functional alterations may directly reflect and potentially influence pathological changes in the brain. Building on these insights, we propose the diabetic retina–brain axis hypothesis, which posits that diabetes simultaneously impacts retinal and cerebral tissues through shared pathogenic mechanisms, including metabolic dysregulation, neuroinflammation, and barrier disruption. Early retinal changes may serve as valuable biomarkers for assessing and forecasting the onset and progression of diabetic cerebral impairment, highlighting a bidirectional interaction and concurrent pathological development between the two systems. This conceptual framework offers a systematic perspective on the multifactorial pathogenesis of DCI and paves the way for early diagnosis and targeted therapeutic interventions.

## 2 Method

We conducted a comprehensive search in the PubMed database from its inception to November 2025, with no restrictions applied regarding language. The search terms utilized included: Diabetes Mellitus, Diabetic Angiopathies, Diabetic Retinopathy, and Diabetic cognitive impairment.

## 3 Physiological commonalities between the retina and the brain

The BRB of the retina closely resembles the BBB of the brain in structure and function. Therefore, assessing BRB integrity provides a reliable reference for investigating BBB dysfunction. The BBB consists of tight junctions (TJs) of cerebral microvascular endothelial cells, astrocytic end–feet, and supporting pericytes. The BRB comprises an inner barrier formed by retinal vascular endothelial cells and an outer barrier formed by retinal pigment epithelial cells; both layers include glial cells and pericytes (14–16). These barriers primarily regulate transmembrane transport of ions, proteins, and water, while preventing infiltration of circulating immune cells. This regulation maintains a stable internal microenvironment for neural tissue.

Both retina and brain display highly precise neurovascular coupling, which autonomously adjusts local blood flow in response to neuronal activity, thereby matching energy supply to demand (17). In the retina, this process can be monitored non–invasively through fundus imaging, enabling real–time quantitative observation. Because of the regulatory consistency between retina and brain, parameters such as vascular reactivity, blood–flow density, and vascular network morphology observed in the retina provide a distinctive window for assessing cerebral microcirculation (18, 19).

Immune privilege was first demonstrated by studies showing that the survival duration of allogeneic tissues implanted in specific regions, such as the brain, was significantly prolonged compared with other areas (20). As an extension of the CNS, the retina also exhibits immune–privilege characteristics. These are maintained by the BRB, the absence of conventional lymphatic vessels, and the relatively uniform distribution of resident microglia (21). Both retina and brain employ physical barriers and local immunosuppressive microenvironments to limit excessive peripheral immune–cell infiltration, thereby protecting irreplaceable neurons from inflammatory damage. Resident microglia are the primary immune cells in both retina and brain (22). Their activation, morphological changes, and release patterns are closely linked under both homeostatic and pathological conditions.

Thus, the retina shares considerable similarity with the brain regarding tissue architecture, cellular composition, and barrier characteristics. It should be regarded not merely as a simple photoreceptive organ, but as an “external brain tissue.”

## 4 Shared pathological mechanisms of the retina and the brain in diabetes

**Figure 1:**
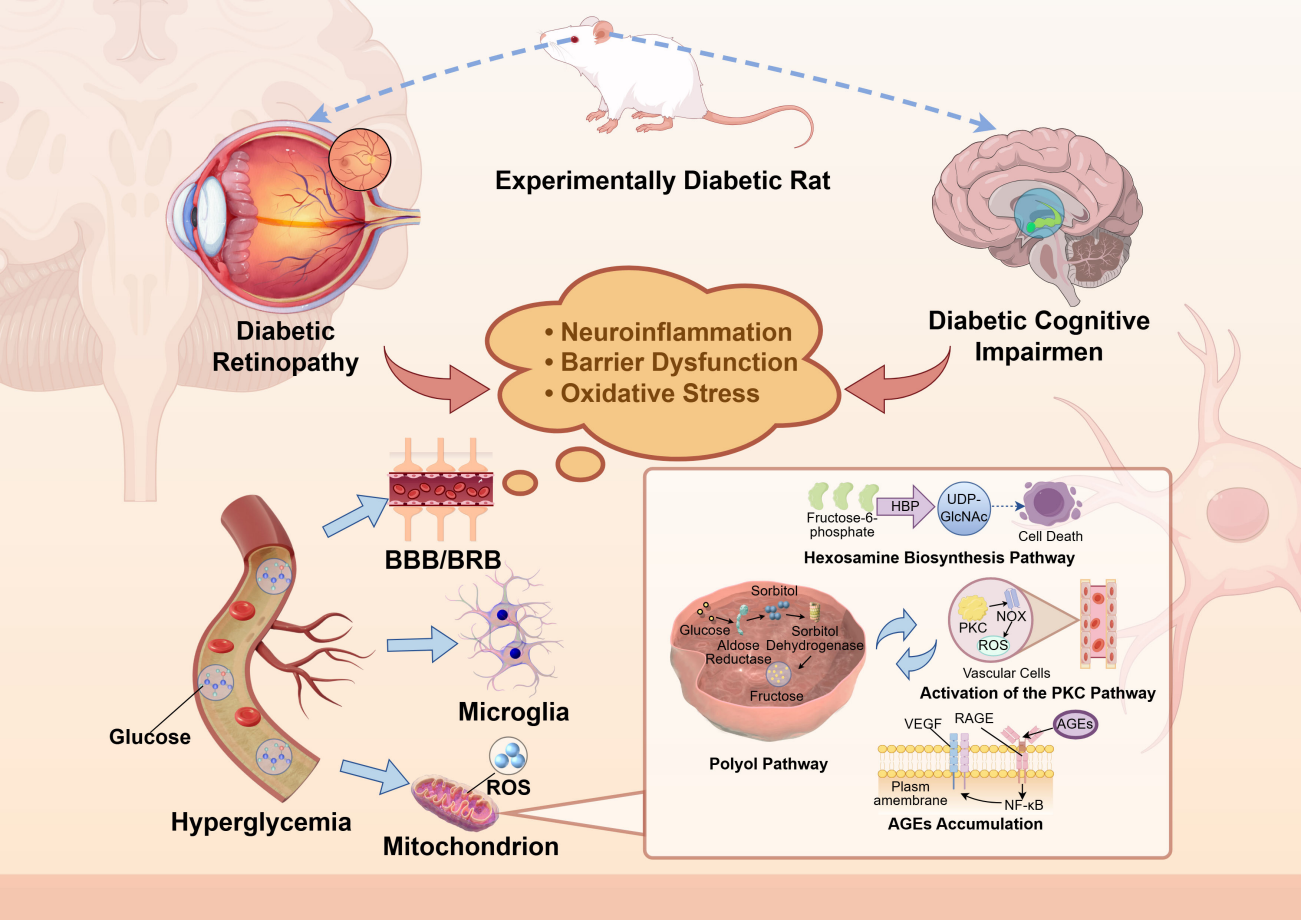
Shared pathological mechanisms of the retina and the brain in diabetes. ROS, reactive oxygen species; HBP, hexosamine biosynthesis pathway; PKC, protein kinase C; NOX, NADPH oxidase; VEGF, vascular endothelial growth factor; RAGE, receptor for advanced glycation end products; AGEs, advanced glycation end products; NF−κb, nuclear factor−κb; UDP−GlcNAc, uridine diphosphate−N−acetylglucosamine.

The retina and brain share a critical vulnerability: high metabolic activity and energy consumption. This metabolic demand renders them uniquely susceptible to hyperglycemic injury. In diabetes, a cascade of pathologies—including neuroinflammation, oxidative stress, AGEs accumulation, and barrier dysfunction—simultaneously assaults both organs. This constellation of shared mechanisms forms the very foundation of the diabetic retina-brain axis hypothesis. These parallel processes do not merely co-occur; they facilitate a damaging crosstalk, creating a vicious cycle of deterioration between the retina and the brain (**Figure 1**).

### 4.1 Neuroinflammation and glial cell activation

Sustained low–grade inflammation is a common feature of diabetic neuropathy, manifesting as chronic inflammatory responses in neural tissues of both the peripheral nervous system (PNS) and the CNS. In the hyperglycemic environment of diabetes, retinal and cerebral glial cells exhibit multilayered activation and functional dysregulation. Key pathogenic factors include alterations of glial cells, functional abnormalities, loss of myelin, and changes in Schwann cells.

In the retina, high glucose activates Müller cells and microglia, leading to the release of cytokines and chemokines. Müller cells are first stimulated by glucose–induced oxidative stress and the polyol pathway, which leads to cellular hypertrophy and up–regulation of glial fibrillary acidic protein (GFAP). In addition, the function of the Kir4.1 potassium channel is reduced, disrupting potassium homeostasis and causing intracellular edema. High glucose also induces excessive expression of CD40 on Müller cells and the release of adenosine–triphosphate (ATP); ATP acts on the P2X7 receptor of microglia, further amplifying inflammatory signaling. Müller cells secrete vascular endothelial growth factor (VEGF), interleukin–1β (IL–1β), tumor necrosis factor–α (TNF–α), and monocyte chemoattractant protein 1 (MCP–1), compromising the BRB and, through NF–κB signaling, inducing pro–inflammatory gene expression, thereby forming a vicious cycle of inflammation and barrier disruption (23).

Similarly, in the brains of diabetic patients, microglia and astrocytes become activated, initiating neuroinflammatory responses (24, 25). In diabetic models, microglia increase in number, adopt a short–branched morphology and a classically activated (M1) phenotype, markedly up–regulating TNF–α, interleukin–6 (IL–6), IL–1β, and other pro–inflammatory cytokines, and further amplifying local inflammation via the Toll–like receptor 4 (TLR4)/NF–κB axis. Under hyperglycemic conditions, elevated levels of fetuin–B (FETUB) directly activate microglial TLR4/NF–κB signaling, leading to phosphorylation, ubiquitination, and proteasomal degradation of inhibitor of κBα (IκBα), nuclear translocation of NF–κB p65, and transcription of inflammatory genes (26). Astrocytes also respond to high glucose with GFAP up–regulation, cellular hypertrophy, and release of pro–inflammatory mediators [IL–6, TNF–α, intercellular adhesion molecule–1 (ICAM–1)]. Their activation likewise depends on IκBα phosphorylation and degradation; NF–κB enters the nucleus and regulates expression of adhesion molecules and chemokines, promoting increased permeability of the BBB (27).

Both cell types share a core inflammatory signaling cascade: ligand binding to receptors such as TNFR1 or TLR4 recruits adaptor proteins including TRADD and TRAF2, forming a signaling complex that activates the IκB kinase (IKK) complex. IKKβ phosphorylates IκBα, which is then ubiquitinated and degraded by the proteasome, removing inhibition on NF–κB. The released p65/p50 dimer translocates to the nucleus, binds κB elements, and initiates transcription of inflammatory genes. Cytokines, adhesion molecules, and chemokines such as TNF–α, IL–1β, IL–6, and ICAM–1 are subsequently up–regulated (28). NF–κB–mediated downstream effects not only directly damage neurons but also disrupt the integrity of the BRB and BBB, increasing barrier permeability and further aggravating retinal neurodegeneration and cognitive decline.

### 4.2 Dysfunction of the BRB and BBB

Hyperglycemia damages all cellular components of the neurovascular unit through multiple molecular pathways. Endothelial cells exposed to hyperglycemia show increased transcytosis, reduced expression of tight–junction proteins such as claudin–5 and ZO–1, and decreased nitric–oxide bioactivity. These changes raise the permeability of the BBB (29). Simultaneously, the glycocalyx becomes thinner and its volume decreases, weakening its barrier function against plasma proteins and leukocytes and promoting microvascular leakage (30, 31).

Pericytes undergo extensive apoptosis or detach from the basement membrane in diabetes, losing their ability to regulate capillary diameter. Recent studies demonstrate that pericytes release ATP via Pannexin 1 (Panx1) to control local Ca^2+^ signals, which in turn regulate capillary constriction and dilation. Loss of pericytes or inhibition of Panx1 results in persistent capillary constriction and impaired dilation, impairing blood–flow regulation and reducing clearance of metabolic waste (32). Moreover, pericyte dysfunction further compromises BBB integrity, allowing harmful metabolites and inflammatory factors to infiltrate neural tissue. Astrocyte end–feet retract, glucose–transporter expression declines, and pro–inflammatory cytokine release increases under hyperglycemia. These alterations weaken astrocytic support of TJs and exacerbate neuronal oxidative stress (33). Neurons suffer from disrupted glucose metabolism, accumulation of reactive–oxygen species, and the actions of AGEs, leading to synaptic dysfunction and apoptosis, which further disrupt neuro–vascular coupling (34).

Barrier dysfunction is a major pathogenic factor in both DR and DCI. The BBB is a highly selective system that tightly regulates exchange between blood and brain. Neuroinflammation and oxidative stress are critical contributors to BBB damage; they down–regulate tight–junction protein expression and compromise cellular junction integrity (35). A prospective study showed that pre–diabetic Lepr db/db mice already exhibit significant BBB leakage (36). Diabetes alters the expression and distribution of tight–junction–associated proteins, ultimately leading to barrier dysfunction (37). Similarly, the BRB tightly controls transport of proteins, ions, and water to maintain retinal neuronal homeostasis (38). In DR, BRB disruption has been consistently demonstrated in experimental and clinical studies (39, 40). Diabetes–induced pericyte loss, intercellular–junction impairment, and basement–membrane thickening exacerbate BRB dysfunction, mirroring the damage observed in the BBB (41, 42).

### 4.3 Oxidative stress and mitochondrial dysfunction

#### 4.3.1 Oxidative stress

Oxidative stress, defined by excessive production of reactive oxygen species (ROS) and weakened antioxidant defenses, reduces the capacity to eliminate ROS. Extensive research has established oxidative stress as a pivotal factor in the pathogenesis of DR and related brain injury (43). In high–glucose–induced retinopathy, oxidative damage involves four well–known metabolic abnormalities: protein kinase C (PKC) pathway activation, increased polyol pathway flux, hexosamine pathway activation, and accumulation of intracellular AGEs (44, 45). Comparable mechanisms occur in the brain, where hyperglycemia induces mitochondrial dysfunction, leading to reduced ATP production, impaired fatty–acid β–oxidation, and elevated ROS levels (46). These changes cause lipid, protein, and DNA oxidation, resulting in neuronal loss, reduced dendritic spine density, and impaired synaptic plasticity. Studies indicate that in type 2 diabetes, mitochondrial dysfunction and altered glucose–metabolism rates in frontal, temporal–parietal, and cingulate regions are key contributors to cognitive decline (47).

Activation of the Polyol Pathway – Hyperglycemia activates the polyol pathway, increasing sorbitol accumulation and ROS production. Sorbitol, being highly hydrophilic, raises intracellular osmotic pressure, causing hypertonic stress, abnormal capillary permeability, and cell apoptosis in retinal and cerebral vessels. Fructose generated in this pathway can be phosphorylated to fructose–3–phosphate and further to 3–deoxyglucosone, both precursors of AGEs (48, 49). To compensate for NADPH consumption, the glucose–6–phosphate shunt is up–regulated, reducing cofactors needed for glutathione (GSH) synthesis and diminishing antioxidant capacity, thereby disrupting redox homeostasis.

Disruption of the Hexosamine Pathway – Clinical studies have confirmed that hexosamine levels are markedly elevated in retinal and brain tissues of diabetic patients. Elevated ROS inhibit glycerol–3–phosphate dehydrogenase (GAPDH), diverting fructose–6–phosphate into the hexosamine biosynthesis pathway. This leads to production of uridine diphosphate–N–acetylglucosamine (UDP–GlcNAc) (50). UDP–GlcNAc serves as a substrate for O–linked N–acetylglucosamine (O–GlcNAc) modification of serine and threonine residues. Recent work shows that high–glucose–induced O–GlcNAc modification of NF–κB promotes retinal ganglion–cell apoptosis (51). Moreover, increased glucosamine from hexosamine pathway activation enhances mitochondrial ROS production and impairs respiratory function, aggravating neuronal insulin resistance and synaptic plasticity (52–54).

Activation of PKC – Aberrant PKC activation is a major pathogenic mechanism in diabetic neuropathy. Hyperglycemia elevates diacylglycerol (DAG) synthesis, which acts as an endogenous PKC activator. Persistent DAG accumulation in diabetes leads to excessive PKC signaling. This impairs vascular regulation and barrier integrity. In retinal and cerebral tissues, PKC regulates BRB and BBB hemodynamics, endothelial permeability, leukocyte adhesion, and VEGF expression (55–57). PKC also activates NADPH oxidase (NOX), increasing ROS production in endothelial cells, smooth–muscle cells, pericytes, and mesangial cells (58). Thus, high glucose stimulates PKC signaling, intensifying oxidative stress.

Formation of AGEs – Chronic hyperglycemia leads to non–enzymatic glycation of proteins, lipids, and nucleic acids, forming AGEs. AGE–induced ROS generation is central to DR and DCI pathogenesis. AGEs accumulate on long–lived matrix proteins such as collagen, increasing arterial stiffness, endothelial dysfunction, and disrupting extracellular–matrix interactions (59, 60). Interaction of AGEs with the receptor for advanced glycation end products (RAGE) activates signaling pathways including MAPK/ERK, TGF–β, JNK, and NF–κB, exacerbating oxidative stress and inflammation (61–63). RAGE–mediated NF–κB activation leads to pericyte apoptosis and VEGF up–regulation, ultimately increasing vascular permeability and damaging both BRB and BBB (64).

The RAGE–mediated signaling pathway directly activates NOX, establishing a pathogenic positive–feedback loop, the AGE–RAGE–NOX–ROS axis (65). Experimental evidence shows that AGE binding to RAGE up–regulates the catalytic NOX subunit gp91^phox^ in various cell types, such as human endothelial cells, retinal pericytes, and neurons. This up–regulation boosts NOX activity, increasing superoxide (O_2_•^–^) production, which is then dismutated into hydrogen peroxide (H_2_O_2_) and hydroxyl radicals, markedly raising intracellular ROS levels (66). Importantly, in vitro studies show that the entire cascade is blocked by the NOX–specific inhibitor diphenyleneiodonium (DPI), confirming NOX as the central driver of AGE–RAGE–induced oxidative stress (67). The accumulated ROS activate key transcription factors such as NF–κB and AP–1, inducing the expression of pro–inflammatory cytokines (e.g., TNF–α, IL–1β, IL–6) and the profibrotic factor TGF–β. This process fosters a sustained inflammatory microenvironment and exacerbates vascular permeability (68). Thus, the AGE–RAGE axis, through direct NOX activation, represents a central mechanism underlying the oxidative stress and chronic inflammation that characterize diabetic microvascular complications.

#### 4.3.2 Mitochondrial dysfunction

Chronic hyperglycemia drives glucose metabolism toward the tricarboxylic acid (TCA) cycle, causing excess accumulation of the electron donors NADH and FADH_2_. This overload increases the burden on the mitochondrial electron–transport chain (ETC). Electron leakage from complexes I and III rises markedly, generating large amounts of O_2_•^–^ that are subsequently dismutated to H_2_O_2_, thereby establishing an oxidative stress environment (69, 70). Under these conditions the mitochondrial inner–membrane potential depolarizes, evidenced by a reduced ΔΨm and heterogeneous fluorescence signals, indicating collapse of the proton gradient (71). Depolarization is accompanied by mitochondrial Ca^2+^ overload, triggering opening of the permeability–transition pore (mPTP). This permits release of cytochrome c, apoptosis–inducing factor (AIF), and additional pro–apoptotic proteins into the cytosol, activating the caspase–9/–3 cascade and initiating intrinsic apoptosis (72).

Hyperglycemia–induced ROS and altered phosphorylation signaling pathways up–regulate the fission–related proteins Drp1 and Fis1, promoting mitochondrial fragmentation. Fusion proteins, such as Mfn2 and OPA1, are down–regulated, limiting mitochondrial fusion (73). Excessive fission impairs mitophagy, thereby diminishing clearance of damaged mitochondria. Consequently, damaged mitochondria accumulate in neurons and retinal cells, continuously releasing ROS and apoptotic signals. This leads to energy deficiency, synaptic dysfunction, and apoptotic death of retinal ganglion cells (RGCs) (74). Together, metabolic reprogramming and the ensuing energy crisis constitute a core molecular mechanism underlying DR and diabetic neurodegeneration.

Moreover, insulin resistance exacerbates mitochondrial dysfunction, oxidative stress, and neuronal apoptosis in the brain (75), and it also promotes the formation of Aβ plaques and the hyperphosphorylation of tau protein, ultimately leading to cognitive decline (76). In addition, studies have demonstrated that an elevated TyG index increases the risk of developing diabetic retinopathy (77). Consequently, hyperglycemia induces concurrent retinal and cerebral damage through oxidative stress, neuroinflammation, barrier disruption, and metabolic disturbances. These interrelated mechanisms form a progressively worsening pathological system, providing a robust foundation for understanding the close relationship between DCI and retinal lesions and for developing therapeutic strategies that target the retina–brain axis.

## 5 Multidimensional verification of the “retina–brain axis” hypothesis in diabetes

Based on this theoretical framework, we propose the diabetic retina–brain axis hypothesis, which posits that the retina, as an extension of the CNS within the eye, undergoes pathological changes under elevated blood–glucose conditions. These changes not only serve as a “window” into cerebral lesions—reflecting microvascular, neuronal, and glial damage—but may also actively contribute to cognitive decline through shared inflammatory, vascular, and metabolic pathways. Empirical support for this hypothesis comes from clinical epidemiology, neuroimaging, and animal–model research.

### 5.1 Clinical epidemiological studies

Cross–sectional investigations have shown a positive correlation between DR severity and cognitive impairment risk. The prevalence of mild cognitive impairment (MCI) is significantly higher in patients with DR, and cognitive decline worsens as DR progresses (78). Individuals with proliferative diabetic retinopathy (PDR) exhibit lower Montreal Cognitive Assessment (MoCA) scores than those with non–proliferative diabetic retinopathy (NPDR). Multivariate logistic regression confirms that DR is an independent risk factor for cognitive dysfunction in type 2 diabetes. A prospective cohort of 682 diabetic patients followed for an average of four years found a markedly higher dementia incidence in the DR group (79). Multivariate analysis revealed that participants with moderate–to–severe DR had a three–fold higher risk of cognitive impairment compared with those without DR. These data underscore a strong association between DR and dementia, supporting the retina–brain axis concept.

### 5.2 Retinal biomarkers

Non–invasive retinal imaging techniques provide valuable biomarkers for assessing CNS status. Structural, functional, and vascular retinal measurements correlate significantly with brain abnormalities and cognitive decline. Optical coherence tomography (OCT) and fundus photography enable detailed retinal examination, making the retina a promising source of biomarkers for diabetes–related cognitive impairment. Evidence suggests that retinal parameters can partially reflect specific brain pathology and facilitate early detection of cognitive decline before substantial clinical symptoms appear. However, the specificity of these retinal changes for DCI, as opposed to other neurodegenerative conditions, remains to be fully elucidated.

#### 5.2.1 Retinal nerve fiber layer thickness

The retinal nerve fiber layer (RNFL) consists of axons from RGCs and serves as an indicator of RGC integrity. RNFL thinning is an early manifestation of DR, occurring before microvascular changes. A similar pattern of ganglion–cell loss is observed in the hippocampus of patients with DCI (80). OCT studies show that RNFL thickness in diabetic patients with cognitive impairment is markedly reduced compared with cognitively normal diabetics (81). For example, a study of 134 type 2 diabetes patients found that macular RNFL and ganglion–cell complex thickness were lower in those with MCI (82). Another investigation of 108 type 2 diabetes patients reported a significant reduction in average RNFL thickness of the right eye in MCI participants, which positively correlated with MoCA and Mini–Mental State Examination (MMSE) scores. Receiver operating characteristic analysis indicated that RNFL thickness is a highly effective diagnostic marker for T2DM–MCI, with an area under the curve of 0.853 and a sensitivity of 91.7 % (83). The above findings demonstrate that RNFL thickness is a sensitive and specific diagnostic marker for diabetic cognitive impairment. However, this conclusion still requires verification through prospective, multicenter trials.

#### 5.2.2 Macular microvascular changes

The macula, essential for central vision, contains a dense microvascular network that is highly susceptible to hyperglycemia. Optical coherence tomography angiography (OCTA) visualizes retinal microvasculature non–invasively. Studies indicate that diabetic patients with cognitive impairment exhibit reduced vessel density in superficial and deep capillary plexuses, enlarged foveal avascular zone (FAZ), and microaneurysms (84–86). An investigation of 100 type 2 diabetes patients showed that those with MCI presented with decreased perfusion density in both capillary layers, abnormal central foveal thickness, and enlarged FAZ area. While no single microcirculation indicator alone showed a statistically significant association with MCI, combined analysis of four indicators revealed a highly significant relationship (p < 0.001) (85). These findings suggest that comprehensive OCTA microcirculation assessment can elucidate the link between retinal microvascular dysfunction and MCI in diabetes.

#### 5.2.3 β–Amyloid deposition

β–Amyloid (Aβ) aggregation is a hallmark of Alzheimer’s disease. Emerging evidence shows that Aβ can also accumulate in the retina of diabetic patients with MCI (87, 88). Retinal Aβ can be detected using fluorescent probes based on curcumin derivatives or intrinsic fluorescence imaging. In diabetic individuals, retinal Aβ levels correlate with hippocampal Aβ accumulation and cognitive impairment (89, 90). Preclinical studies using diabetic rabbit models have demonstrated a causal relationship between retinal and brain Aβ deposition and associated cognitive deficits (91). Thus, retinal Aβ may serve both as a biomarker and a pathological mediator in diabetes–related cognitive impairment.

### 5.3 Preclinical studies

Similar pathological alterations in retina and brain, especially in hippocampal subregions and cortical layers, have been documented in streptozotocin (STZ)–induced diabetic rats, db/db mice, and zebrafish models (92, 93). Core mechanisms include basement–membrane thickening of retinal and cerebral microvessels, pericyte loss, and tight–junction disruption, leading to BRB and BBB compromise. Activation of Müller glial cells, microglia, and astrocytes releases pro–inflammatory cytokines such as TNF–α, IL–1β, and IL–6, sustaining chronic neuroinflammation. Apoptosis of RGCs and hippocampal neurons, along with impaired synaptic plasticity, is observed. These processes are mediated by oxidative stress, mitochondrial dysfunction, reduced brain–derived neurotrophic factor (BDNF) levels, and an imbalanced glutamate/GABA ratio, leading to excitotoxicity. Importantly, even after blood–glucose control, neurodegenerative changes may persist, indicating a “metabolic memory” effect (94).

## 6 Therapeutic insights and future directions

**Figure 2:**
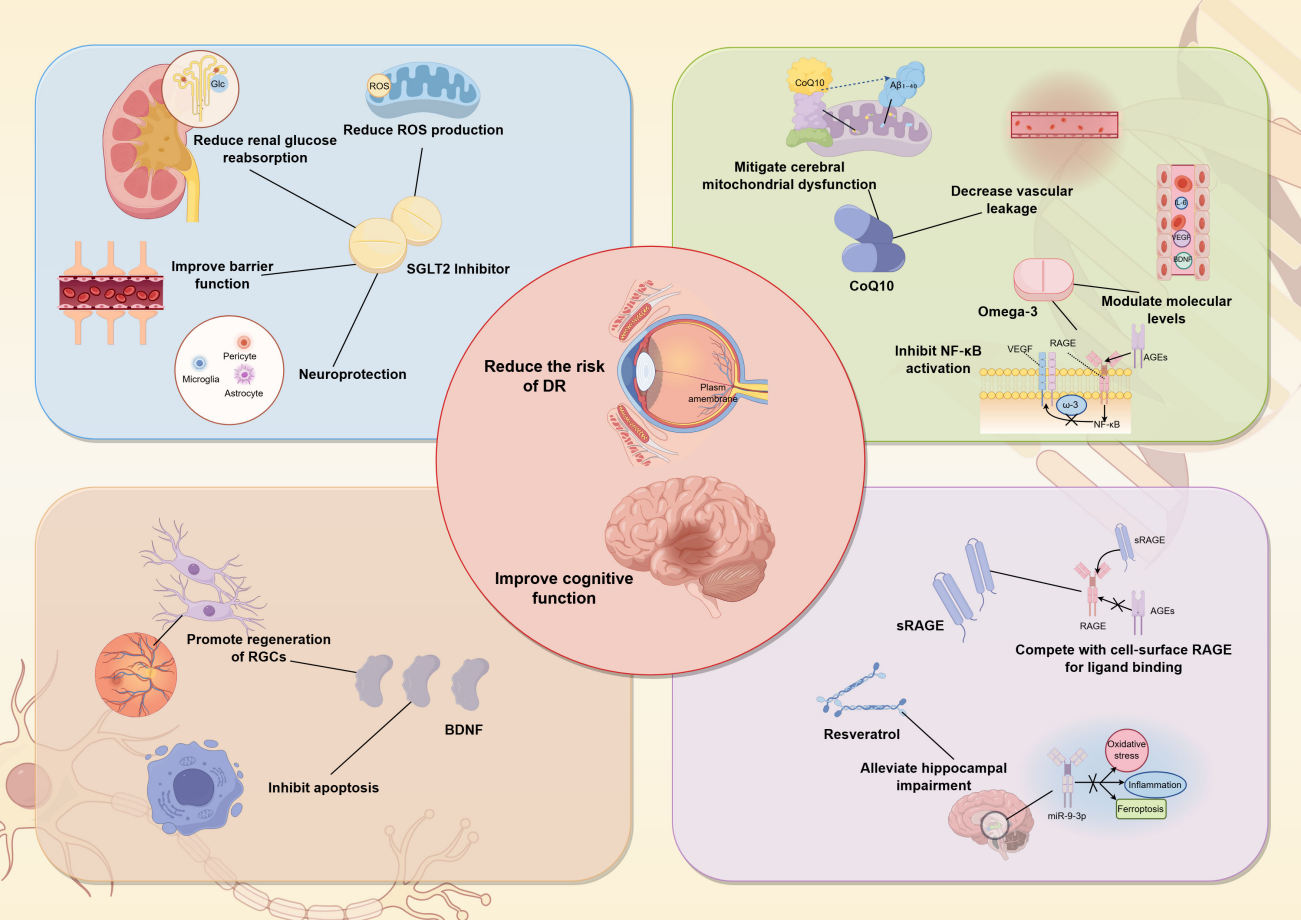
Novel synchronous therapeutic for DCI and DR. SGLT2, sodium−glucose cotransporter ;2; Glc, glucose; ROS, reactive oxygen species; VEGF, vascular endothelial growth factor; RAGE, receptor for advanced glycation end products; CoQ10, Coenzyme ;Q10; RGCs, retinal ganglion cells; BDNF, brain−derived neurotrophic factor; miRNA, MicroRNA; AGEs, advanced glycation end products; sRAGE, soluble receptor for advanced glycation end products.

The retina-brain axis framework fundamentally expands our arsenal against DCI. Here, the retina plays a dual role: it is both a diagnostic portal and a potential therapeutic interface. The clinical advantage is clear. Its accessibility to direct, non-invasive observation makes the retina an unparalleled organ for both screening and monitoring cognitive decline in diabetic patients (**Figure 2**).

### 6.1 Retina–based screening and monitoring of DCI

In clinical practice, existing diabetic fundus–screening programs can be leveraged to assess retinal lesions and identify high–risk populations for cognitive impairment simultaneously. Advances in artificial–intelligence–driven retinal image analysis—based on deep–learning algorithms—hold promise for early DCI detection and risk stratification. These techniques evaluate vascular morphology, RNFL thickness, and microvascular abnormalities, providing a cost–effective, large–scale screening method for at–risk individuals.

### 6.2 therapeutic strategies targeting the retina–brain axis

#### 6.2.1 Hypoglycemic agents

Hypoglycemic drugs remain the cornerstone of diabetes management and may mitigate multi–system damage caused by hyperglycemia. Several agents have demonstrated protective effects on both retina and CNS.

SGLT2 inhibitors reduce renal glucose reabsorption. Beyond cardiovascular and renal benefits, they appear to ameliorate DR and cognitive decline. Mechanisms include reduction of oxidative stress and improvement of barrier function (95–97). Subsequent studies show that SGLT2 inhibitors enhance endothelial function, inhibit pathological remodeling, and protect the BRB, BBB, and neurovascular unit (98). They also suppress acetylcholinesterase activity, contributing to cognitive improvement. In a combined mouse model of diabetes and Alzheimer’s disease, empagliflozin increased BDNF levels, modulated neural transmission, and reduced cerebral microvascular lesions and cognitive impairment (99). Thus, SGLT2 inhibitors hold considerable promise for attenuating the progression of diabetic retinopathy and cognitive impairment.

Glucagon–like peptide–1 (GLP–1) receptor agonists have shown promise in treating DR and cognitive impairment. Clinical data indicate that GLP–1 receptor agonists significantly lower the risk of advanced DR compared with insulin (100). Preclinical studies reveal anti–inflammatory, neurotrophic, and neuroprotective properties of GLP–1 receptor agonists (101). Recent experiments demonstrate that liraglutide mitigates aluminum–induced neurotoxicity, preserves learning ability, and improves spatial memory in rat models of aluminum–induced dementia (102). Epidemiological evidence suggests that GLP–1 receptor agonists may delay dementia progression in diabetic patients (103).

#### 6.2.2 Anti–inflammatory and antioxidant agents

Neuroinflammation and oxidative stress are pivotal in diabetic complications, making anti–inflammatory and antioxidant drugs attractive candidates for DR and DCI.

Coenzyme Q10 (CoQ10) is a lipid–soluble antioxidant located in the inner mitochondrial membrane, where it transfers electrons from complexes I and II to complex III (104). CoQ10 exhibits both energy–regulating and anti–inflammatory properties, and can inhibit neuronal apoptosis (105). In diabetic rat models, CoQ10 improves oxidative phosphorylation efficiency, suppresses hydrogen–peroxide production, and enhances synaptic plasticity (106). The findings demonstrate that CoQ10 mitigates Aβ_1–40_-induced cerebral mitochondrial dysfunction, implicating its potential for ameliorating the severe energy metabolism impairment common to both diabetic and Alzheimer’s pathology (107). Idebenone, a CoQ10 analogue, reduces oxidative damage, prevents RGC loss, inhibits glial proliferation, decreases vascular leakage, and preserves retinal thickness (108–110).

Omega–3 fatty acids possess antioxidant activity that protects cells from ROS–mediated damage, thereby alleviating diabetes–related clinical symptoms (111, 112). In STZ–induced diabetic rats, omega–3 supplementation can help normalize the levels of VEGF, IL–6, and brain–derived neurotrophic factor (BDNF) in serum and retinal (113). It also inhibits NF–κB activation, down–regulates NOD–like receptor protein ;3 (NLRP3) inflammasome activity, and reduces expression of amyloid precursor protein (APP) and β–secretase ;1 (BACE1), thereby delaying Alzheimer–related cognitive decline (114).

#### 6.2.3 Neuroprotective interventions

Neuroprotective strategies aim to preserve neuronal function in retina and brain, preventing neurodegeneration and cognitive decline.

BDNF is a key neurotrophin essential for neuronal development and maintenance. In addition to being highly expressed in the human brain, BDNF is also synthesized by retinal neurons and glial cells (115). In the retina, BDNF inhibits apoptosis and promotes regeneration of RGCs. Studies demonstrate that intervention with an appropriate dose of BDNF during the early stages of retinopathy delays the progression of neuroretinal degeneration and downregulates the expression of VEGF (116, 117). Clinical observations indicate that serum BDNF levels in patients with type 2 diabetes accompanied by cognitive impairment are significantly lower than those without cognitive impairment, exhibiting a persistent downward trend (118). Animal studies indicate that up–regulating BDNF ameliorates cognitive impairment in diabetic mice (119).

#### 6.2.4 Emerging targeted therapies

RAGE inhibitors – The AGE–RAGE signaling pathway plays a central role in retinal and cerebral damage. Soluble RAGE (sRAGE) competes with cell–surface RAGE for ligand binding, facilitating clearance or neutralization of circulating ligands. Elevated plasma sRAGE levels are associated with reduced risk of cognitive impairment and Alzheimer’s disease, making sRAGE–enhancing strategies promising for vascular and neurodegenerative protection (120). In DR, hyperglycemia up–regulates RAGE expression in retinal cells, activating pro–oxidative and pro–inflammatory pathways (121). Preclinical studies demonstrate that AGE inhibitors mitigate retinal and neurological lesions (122).

MicroRNA (miRNA) therapies – miRNAs regulate gene expression post–transcriptionally (123). In diabetes, dysregulated miRNA expression in retinal and cerebral tissues contributes to pathology. Modulating miRNAs can concurrently alleviate retinal damage and cognitive deficits (124). Resveratrol up–regulates miR–21, restoring insulin signaling and improving memory in diabetic rat models (125). It also reduces exosome–derived miR–9–3p, alleviating oxidative stress, inflammation, and ferroptosis in the hippocampus, thereby providing cognitive protection (126). Clinical strategies focus on nuclease–protected miRNA mimics (miR–mimics) or inhibitors (anti–miRs), and exosome–mediated delivery to the eye, enabling precise therapeutic intervention (127). Due to their ability to precisely regulate disease-associated pathways, miRNAs have emerged as attractive therapeutic targets and intervention tools.

## 7 Discussion

DCI represents a primary manifestation of CNS complications in diabetes. Early detection and effective intervention remain challenging. The diabetic retina–brain axis hypothesis offers novel perspectives for systematically elucidating DCI pathogenesis and for clinical prevention and management. The hypothesis rests on the high homology between retina and brain in embryonic origin, anatomy, and physiology, proposing that oxidative stress, neuroinflammation, and barrier dysfunction concurrently affect retinal and cerebral tissues, leading to parallel damage.

Numerous clinical studies have consistently shown that RNFL thinning, ganglion–cell layer thinning, reduced microvascular density, and β–amyloid deposition correlate with brain structural degeneration and cognitive decline. These retinal biomarkers not only serve as diagnostic criteria for retinal lesions but also provide valuable tools for early detection and dynamic monitoring of cognitive decline.

Therapeutically, interventions targeting the common pathway of the retina–brain axis have dual potential. For example, SGLT2 inhibitors delay DR progression while inhibiting neuroinflammation and preserving BBB integrity. Antioxidants such as CoQ10 mitigate oxidative stress in both retina and CNS, reducing tissue damage. Neurotrophic factor replacement and certain targeted pharmacological agents may synergistically protect retinal and neural tissues.

Despite its compelling logic, translating the diabetic retina-brain axis hypothesis into clinical practice confronts significant hurdles.① The hypothesis posits that DCI and DR share common pathogenic mechanisms, thereby explaining their association. However, whether DR directly precipitates cognitive decline and the causal relationship between the two conditions remain unresolved.② Most existing evidence is derived from observational studies, lacking RCTs that can establish causality. Small–sample investigations have reported positive correlations between retinal vascular density, retinal layer thickness, and the risk of cognitive impairment, yet these biomarkers have not been uniformly evaluated in large–scale, multicenter prospective cohorts; consequently, their predictive value and clinical feasibility are still uncertain.③ Preliminary data suggest that improvement of visual function may accompany cognitive enhancement, but this conclusion is based on limited samples and requires confirmation through rigorously designed RCTs. Future research should therefore prioritize: elucidating the precise mechanistic pathways linking inflammation, vascular dysfunction, and neural interactions within the retina-brain axis; validating retinal biomarkers in extensive, multicenter prospective cohorts to identify high-risk populations; and (conducting high-quality, large-sample RCTs to assess the causal impact of targeted interventions. This systematic approach will enable robust evaluation of retina-brain axis-directed therapies for protecting cognitive function in patients with diabetes.
